# Cytotoxic Mechanism of Deep-Sea Fungus *Chaetomium globosum* YP-106 Metabolite Chaetomugilin O in Thyroid Cancer Cells

**DOI:** 10.3390/md23100370

**Published:** 2025-09-24

**Authors:** Yaqin Fan, Wenhui Xiong, Yuting Qiu, Yang Li, Xin Liu, Peiqing He, Guian Huang

**Affiliations:** 1 School of Life Sciences, Qingdao Agricultural University, Qingdao 266109, China; fanyaqin@qau.edu.cn (Y.F.); xiong0215nn@163.com (W.X.); qyutingsw2025@163.com (Y.Q.); 19161021606@163.com (Y.L.); liuxin202312@163.com (X.L.); 2 MNR Key Laboratory of Marine Eco-Environmental Science and Technology, First Institute of Oceanography, Ministry of Natural Resources, Qingdao 266061, China; hepeiqing@fio.org.cn

**Keywords:** deep-sea fungus, thyroid cancer, *Chaetomium globosum*, transcriptomic, molecular docking

## Abstract

This study investigated the potential of the deep-sea-derived fungal metabolite, chlorinated azaphilone compound chaetomugilin O, in the treatment of thyroid cancer. Chaetomugilin O was extracted from the fungus *Chaetomium globosum* YP-106 and subjected to in vitro experiments. The results demonstrated that this compound significantly inhibited the proliferation of thyroid cancer CAL-62 cells in a dose-dependent manner, with an IC_50_ value of 13.57 µM. Further mechanistic studies revealed that chaetomugilin O exerts its antitumor effects by inducing reactive oxygen species (ROS) accumulation, G2/M phase cell cycle arrest, and apoptosis. Transcriptomic analysis indicated its regulatory role in the PI3K-Akt signaling pathway, suggesting a multi-target synergistic antitumor mechanism. Molecular docking confirmed that chaetomugilin O binds to the Akt protein, forming a hydrogen bond with Lys158, implying its potential to directly inhibit Akt activity and interfere with PI3K-Akt pathway function. This study provides experimental evidence for the development of novel, low-toxicity, highly effective therapeutic agents for thyroid cancer.

## 1. Introduction

Globally, thyroid cancer is the ninth most prevalent malignant tumor, comprising roughly 3.0% of all newly diagnosed cancer cases. In recent years, the incidence of thyroid cancer has shown a continuous upward trend, with over 500,000 new cases reported annually worldwide, and the incidence rate in women being up to three times higher than that in men [1,2]. Despite the notable advancements in the early detection and management of thyroid cancer, effective therapies for intermediate and advanced-stage patients remain lacking [3]. Meanwhile, traditional chemotherapy drugs, while capable of partially inhibiting tumor growth, suffer from narrow therapeutic windows, significant side effects, and a high propensity to induce drug resistance [4,5]. Therefore, the development of novel, highly effective, and low-toxicity anti-thyroid cancer drugs is urgently needed.

**Figure 1:**
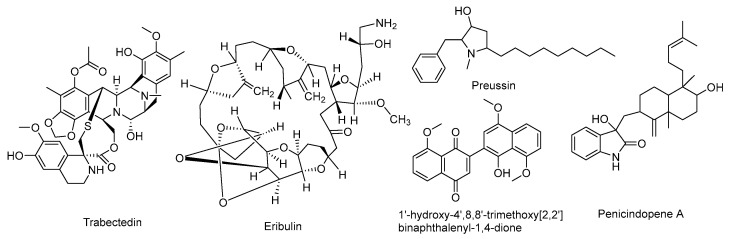
Examples of marine-derived antitumor bioactive compounds.

Constituting 75% of the Earth’s surface area and sustaining over 80% of global biodiversity, oceans represent a fundamental repository for natural bioactive compounds [6]. To adapt to extreme environments characterized by high salinity, high pressure, and other harsh conditions, marine organisms have evolved unique metabolic pathways that produce structurally novel and mechanistically diverse bioactive substances [7,8]. Up to the present day, a number of marine-derived bioactive agents have received marketing authorization for clinical indications, particularly in cancer treatment. Notable examples include Trabectedin (Figure 1), a tunicate *Ecteinascidia turbinata*-derived isolate with clinical approval for soft tissue sarcoma, as well as Eribulin, a sponge-derived Halichondrin B synthetic derivative indicated for metastatic breast cancer and liposarcoma. These achievements highlight the central role of marine-derived compounds in the discovery of anticancer pharmaceuticals [9,10,11].

Marine fungi, as key members of marine ecosystems, have emerged as an important source of antitumor bioactive compounds due to their robust secondary metabolic capabilities [12]. Relevant studies have demonstrated that compounds derived from marine fungi exhibit remarkable antitumor potential. For instance, Preussin, produced by the sponge-associated fungus *Aspergillus candidus*, inhibits breast cancer cell proliferation by inducing cell death; Penicindopene A, isolated from the deep-sea fungus *Penicillium* sp., shows significant cytotoxicity against lung and cervical cancer cells. 1′-hydroxy-4′,8,8′-trimethoxy[2,2′]binaphthalenyl-1,4-dione, isolated from Hypoxylon rubiginosum, showed potent inhibitory activity against a variety of tumor cell lines [13,14,15]. These compounds function through mechanisms such as regulating apoptosis pathways, arresting the cell cycle, and inhibiting angiogenesis, offering new strategies to overcome tumor drug resistance and improve treatment precision. These advances provide strong impetus for further research on the antitumor potential of marine fungi.

**Figure 2:**
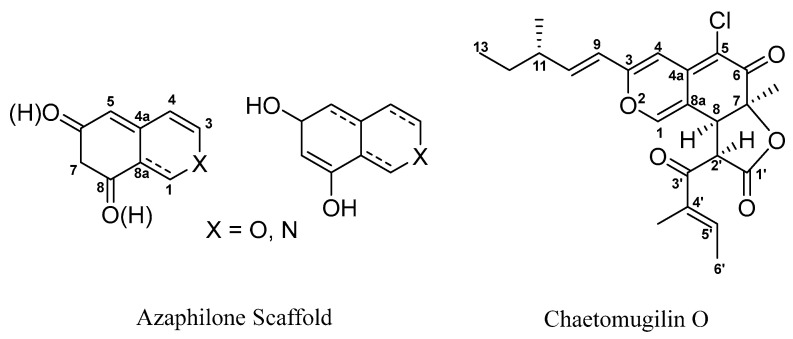
Structures of Azaphilone scaffold and Chaetomugilin O.

Azaphilones represent a subgroup of fungal polyketide secondary metabolites, distinguished by their structurally distinctive features: a highly oxidized pyranoquinone bicyclic skeleton and a quaternary carbon center (Figure 2) [16]. Their structural novelty and diversity confer a wide range of biological activities, including notable antiviral, antibacterial, anti-inflammatory, and anticancer effects [17,18,19]. Among these, the chlorinated azaphilone derivative chaetomugilin O (Figure 2) has attracted considerable attention due to its remarkable cytotoxic activity. This compound has shown significant inhibitory effects on various tumor cell lines, such as mouse leukemia cells P388/L1210, human oral epidermoid carcinoma cells KB, and human promyelocytic leukemia cells HL-60, highlighting the potential of chaetomugilin O in antitumor drug development [20,21].

*Chaetomium globosum* is a deep-sea fungal species with significant research potential, yet the mechanisms and therapeutic value of its metabolites in the treatment of thyroid cancer remain to be fully elucidated. This study focuses on the *C. globosum* strain YP-106, aiming to investigate the anti-thyroid cancer activity of its metabolite chaetomugilin O and explore its underlying mechanisms of action. The research provides experimental evidence for the development of novel targeted chemotherapeutic agents with low toxicity and high selectivity while also offering new biological insights into the antitumor molecular mechanisms of azaphilone compounds in the context of thyroid cancer therapy.

## 2. Results and Discussion

### 2.1. Structure of Chaetomugilin O

The molecular formula of chaetomugilin O, originally isolated as a yellow amorphous powder, was determined to be C_23_H_25_ClO_5_ by high-resolution fast atom bombardment mass spectrometry based on the observed exact mass of the [M + H]^+^ ion and the MH^+^/[MH + 2]^+^ isotope ratio, yielding a formula corresponding to 11 degrees of unsaturation. The ^1^H NMR and ^13^C NMR spectra displayed characteristic signals for 9 quaternary carbons (including 2 carbonyl carbons and 5 olefinic carbons), 8 methine carbons (containing 5 olefinic carbons), 1 methylene group, and 5 methyl groups (Figure 2). The NMR data matched well with literature values for chaetomugilin O, and the optical rotation value ([α]D^25^-103.4) was consistent with the reported value ([α]D^25^-116.1), confirming the identity of the compound as chaetomugilin O [22].

Chaetomugilin O: Yellow amorphous powder; [α]D^25^-104.3 (c 0.10, EtOH); UV_λmax_ (MeOH)/nm (log ε): 230 (5.29), 266 (4.52), 299 (5.01), 339 (4.67), 408 (5.31), 428 (5.08), 436 (5.00) and 455 (2.83) nm; HRESIMS m/z 417.1433 [M + H]^+^ (calcd for C_23_H_25_ClO_5_: 417.1468) (Figure S1); ^1^H and ^13^C NMR data (Figures S2 and S3) are listed in Table 1.

### 2.2. Chaetomugilin O Inhibits CAL-62 Cell Viability Through ROS-Mediated Oxidative Stress

To investigate the effect of the marine fungus-derived compound chaetomugilin O on thyroid cancer, the CCK-8 assay was used to measure its inhibitory effect on the proliferation of CAL-62 cells (an ATC model). The results demonstrated that chaetomugilin O induced a dose-dependent suppression of cell viability within the concentration range of 2.5–50 μM. The half-maximal inhibitory concentration (IC_50_) was determined to be 13.570 ± 0.170 μM. Furthermore, the positive control drug doxorubicin (Dox) exhibited an IC_50_ value of 0.167 ± 0.023 μM (Table 2). These results indicate that chaetomugilin O effectively blocks tumor cell proliferation at relatively low concentrations.

**Figure 3:**
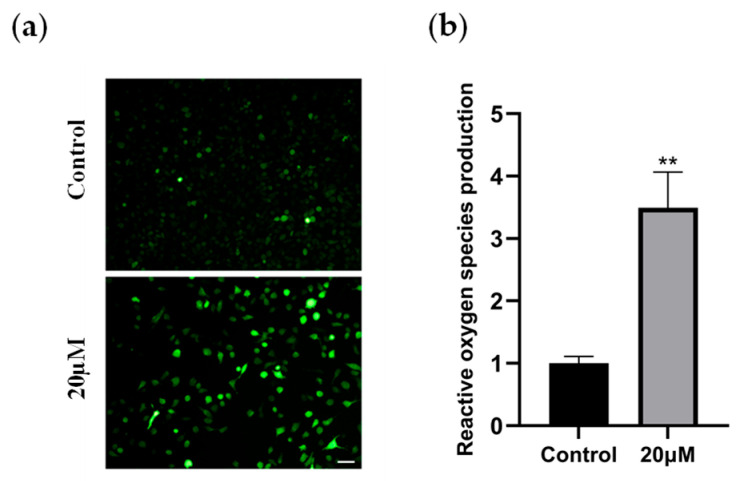
Chaetomugilin O induces ROS accumulation. (**a**,**b**) ROS levels in CAL-62 cells treated with 20 µM Chaetomugilin O were observed under fluorescence microscopy. Scale bar, 50 µm. The results were expressed as mean ± SD of three independent experiments. Compared with the control group, ** *p* < 0.01.

Further studies revealed that intracellular reactive oxygen species (ROS) levels were significantly elevated in the 20 µM treatment group. Fluorescence microscopy showed that compared with the control group, the treated cells exhibited a marked increase in ROS fluorescence signals (Figure 3), suggesting that ROS accumulation may lead to increased mitochondrial membrane permeability and DNA damage, thereby impairing cell survival. This mechanism is closely related to the common oxidative stress defense defects in thyroid cancer, particularly under high metabolic activity, where tumor cells exhibit significantly increased sensitivity to ROS.

These results confirm that chaetomugilin O triggers oxidative damage by inducing ROS accumulation, thereby inhibiting tumor cell viability. This provides an experimental foundation for oxidative stress-targeted therapeutic strategies against thyroid cancer.

### 2.3. Chaetomugilin O Induces G2/M Phase Arrest and Apoptosis in CAL-62 Cells

**Figure 4:**
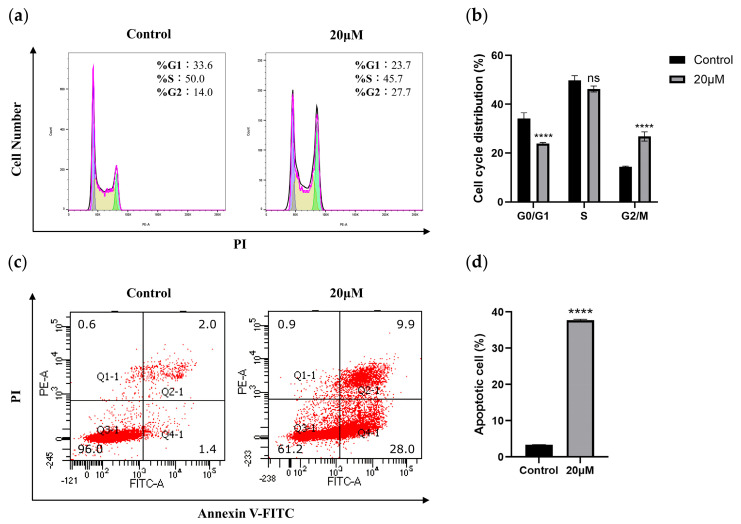
Chaetomugilin O induces G2/M phase arrest and apoptosis in CAL-62 cells. (**a**,**b**) Flow cytometry analysis of cell cycle distribution in CAL-62 cells after 48 h treatment with 20 µM Chaetomugilin O. (**c**,**d**) Apoptosis rates of CAL-62 cells treated with 20 µM Chaetomugilin O for 48 h were determined by flow cytometry. Results were presented as mean ± SD derived from three independent experiments. In comparison with the control group, **** *p* < 0.0001 was observed, with ns representing no statistical significance.

To further investigate the mechanism by which chaetomugilin O inhibits CAL-62 cell viability, we analyzed cell cycle distribution using PI staining combined with flow cytometry. The results showed that after 48 h of treatment with 20 µM chaetomugilin O, the proportion of G2/M phase cells increased from 14.0% to 27.7% compared to the control group (Figure 4a). This indicates that chaetomugilin O can induce G2/M phase arrest in CAL-62 cells, thereby preventing them from entering mitosis.

Cell apoptosis is one of the key pathways through which anticancer drugs exert their effects. To determine whether chaetomugilin O induces apoptosis in thyroid cancer cells, we performed Annexin V-FITC/PI double staining followed by flow cytometric analysis to quantify apoptosis in CAL-62 cells after treatment. The results revealed that only 3.4% of cells underwent apoptosis in the control group (DMSO-treated), whereas treatment with a high concentration of 20 µM chaetomugilin O increased the apoptosis rate to 37.9%, representing an approximately 11-fold increase over the control, demonstrating its significant pro-apoptotic activity (Figure 4b).

These experiments confirm that chaetomugilin O synergistically inhibits thyroid cancer cell proliferation by inducing G2/M phase arrest and apoptosis. The underlying mechanism may involve DNA damage and repair dysregulation: First, chaetomugilin O treatment leads to a significant increase in intracellular ROS levels, which may trigger DNA damage and activate cell cycle checkpoints, thereby inducing G2/M phase arrest. Second, sustained G2/M phase arrest ultimately results in a marked increase in apoptosis, suggesting that failed repair mechanisms may drive cells toward apoptotic pathways. This cascade effect—“cell cycle arrest → repair failure → apoptosis”—may be a crucial mechanism by which chaetomugilin O suppresses thyroid cancer cell proliferation.

These findings highlight chaetomugilin O’s potential in overcoming thyroid cancer resistance, particularly in chemotherapy-insensitive thyroid cancers (as modeled by CAL-62 cells), providing experimental evidence for the development of marine-derived natural products targeting thyroid cancer.

### 2.4. Transcriptomic Analysis Reveals Chaetomugilin O Regulates Cell Cycle and Apoptosis via the PI3K-Akt Pathway

**Figure 5:**
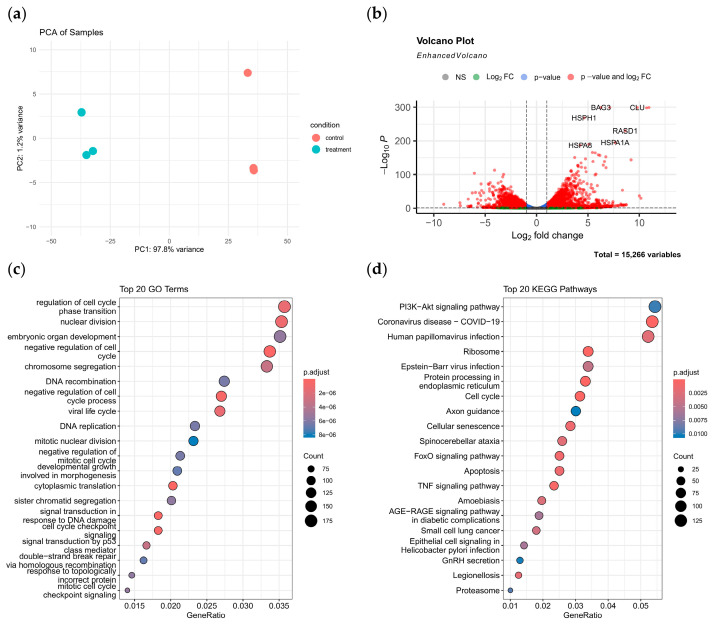
Transcriptomic analysis of CAL-62 thyroid cancer cells treated with chaetomugilin O reveals molecular mechanisms for cell cycle arrest and apoptosis. (**a**) Principal component analysis (PCA) exhibits significant group separation between treated and control samples. (**b**) A volcano plot was used to identify differentially expressed genes (DEGs). (**c**,**d**) Functional annotation via GO and KEGG pathway enrichment analyses highlights the top 20 enriched categories for DEGs.

For the purpose of further investigation into the anti-thyroid cancer mechanism of chaetomugilin O, we performed transcriptomic analysis, which revealed its significant regulatory effects on thyroid cancer CAL-62 cells. Principal component analysis (PCA) demonstrated a clear separation between the treatment group (20 µM chaetomugilin O) and the control group along the first principal component (PC1), with PC1 contributing 97.8% of the variance, indicating that this compound markedly alters the global transcriptomic profile of thyroid cancer cells (Figure 5a). Differential gene analysis identified multiple significantly upregulated genes (Figure 5b), including apoptosis-related genes (BAG3, CLU), heat shock protein family members (HSPA1A, HSPA8, HSPH1), and the stress-responsive protein RASD1, suggesting that chaetomugilin O may exert its anticancer effects by inducing oxidative stress and disrupting apoptotic balance.

Enrichment analysis further elucidated the underlying mechanisms: GO analysis revealed significant enrichment of cell cycle regulation-related terms, such as cell cycle phase transition, mitotic nuclear division, and chromosome segregation, along with DNA damage repair-related terms, including p53-mediated signal transduction (Figure 5c). These findings support the hypothesis that chaetomugilin O inhibits thyroid cancer cell proliferation and promotes apoptosis by interfering with the cell cycle and inducing oxidative stress. KEGG analysis confirmed significant suppression of the PI3K-Akt signaling pathway (Figure 5d), a critical pathway regulating cell survival and proliferation, which may be a key target of the compound. As a key effector molecule of this pathway, Akt (protein kinase B) plays an important role in tumorigenesis by regulating apoptosis, cell cycle, and survival. Besides the PI3K-Akt pathway, apoptosis, cell cycle, and FoxO signaling pathways were also co-enriched, suggesting that chaetomugilin O may exert its effects through a synergistic multi-target regulatory network. These results align with in vitro experimental data (elevated ROS levels, G2/M phase arrest, and increased apoptosis), suggesting that chaetomugilin O likely exerts its anti-thyroid cancer effects through multi-target synergistic mechanisms.

### 2.5. Molecular Docking Analysis

**Figure 6:**
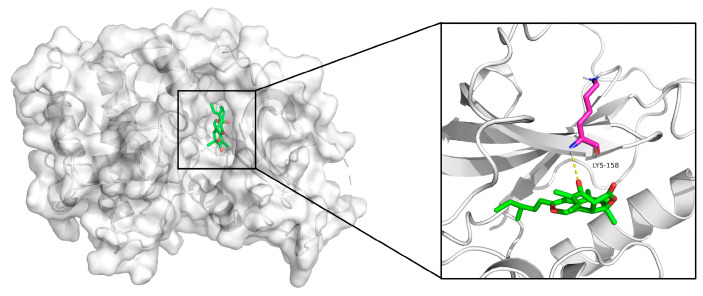
Molecular docking simulation results of compound Chaetomugilin O and Akt.

Given the critical role of Akt in the PI3K-Akt pathway, we performed molecular docking simulations to analyze the interaction between chaetomugilin O and Akt (PDB ID: 4GV1). The results showed that chaetomugilin O stably binds to the active site of Akt with a binding energy of −8.6 kcal/mol, forming a hydrogen bond with the key residue Lys158 (Figure 6). Notably, this binding site significantly overlaps with the interaction region of the known Akt inhibitor AZD5363 [23]. While AZD5363 exerts its inhibitory effect by forming hydrogen bonds with residues such as Ala230, Glu228, Glu234, and Glu278, chaetomugilin O primarily forms a hydrogen bond with Lys158. These findings suggest that chaetomugilin O may occupy the active site of Akt, thereby inhibiting the activation of the PI3K-Akt signaling pathway and exerting potential antitumor effects.

However, it should be noted that the molecular docking results in this study have certain limitations. Although the docking simulations demonstrated favorable binding energy and specific interactions, the method itself is a static simulation that fails to account for conformational changes and binding stability of the protein-ligand complex under dynamic conditions. Therefore, to more comprehensively evaluate the actual binding mode and affinity of chaetomugilin O with Akt, follow-up studies should incorporate molecular dynamics simulations for further validation.

## 3. Materials and Methods

### 3.1. General Experimental Procedures

The optical rotation was measured using a POLAX-L polarimeter (ATAGO, Tokyo, Japan). The ultraviolet (UV) spectrum was recorded with a DU^®^ 640 UV-visible spectrophotometer (Beckman Coulter, Brea, CA, USA). High-resolution electrospray ionization mass spectrometry (HRESIMS) data were acquired using a Micromass EI-4000 (AutospecUltima-TOF) mass spectrometer (Waters Corporation, Milford, MA, USA). Proton nuclear magnetic resonance (^1^H NMR) and carbon-13 nuclear magnetic resonance (^13^C NMR) spectra were determined with a JNM-ECP600 nuclear magnetic resonance spectrometer (JEOL, Tokyo, Japan). For column chromatographic separation, silica gel (100–200 mesh, Qingdao Marine Chemical Co., Ltd., Qingdao, China) and Sephadex LH-20 gel (Pharmacia Biotech, Uppsala, Sweden) were used. Semi-preparative high-performance liquid chromatography (HPLC) was performed on an ODS column (YMC-pack ODS-A, 10 × 250 mm, 5 µm, flow rate: 4.0 mL/min). All organic solvents were purchased from Sinopharm Chemical Reagent Co., Ltd. (Shanghai, China). RPMI 1640 medium and phosphate-buffered saline (PBS) were purchased from Procell Life Science & Technology Co., Ltd. (Wuhan, China). Fetal bovine serum (FBS) was sourced from Biological Industries (Beit HaEmek, Israel). Penicillin-streptomycin solution (dual antibiotic), dimethyl sulfoxide (DMSO), and doxorubicin hydrochloride (DOX) were purchased from Solarbio Life Sciences (Beijing, China). The CCK-8 assay kit was acquired from Biosharp Life Sciences (Hefei, China). Trypsin cell digestion solution (without EDTA), DCFH-DA fluorescent probe, propidium iodide (PI) staining solution, ribonuclease A (RNase A), and the Annexin V-FITC/PI apoptosis detection kit were obtained from Thermo Fisher Scientific (Milwaukee, WI, USA).

### 3.2. Fungal Material

The fungal strain YP-106 was isolated in 2017 from seawater samples collected at a depth of 6215 m in the Yap Trench of the Pacific Ocean. ITS sequencing identified the fungus as *Chaetomium globosum*, showing 99.6% identity with the ITS sequence of *C. globosum* (GenBank accession no. KC202936.1). The sequence of YP-106 has been deposited in GenBank under accession number OL872214. This strain is preserved at the Shandong Provincial Key Laboratory of Applied Mycology, College of Life Sciences, Qingdao Agricultural University, China.

### 3.3. Fermentation, Extraction and Isolation

The fungal strain *C. globosum* YP-106 was initially inoculated onto a revival medium (weight ratio of water/potato/glucose/agar/sea salt = 100:20:2:2:3.3, pH 6.5) and cultured at 28 °C for 3 days. Subsequently, the activated culture was transferred to a seed medium (water/potato/glucose/sea salt = 100:20:2:3.3, pH 6.5) and incubated at 28 °C with shaking at 150 rpm for another 3 days. For large-scale fermentation, a liquid culture medium was prepared (water/malt extract/peptone/sea salt = 100:1.7:3:3.3, pH 6.5), with 300 mL aliquots dispensed into 1 L Erlenmeyer flasks, each inoculated with 10 mL of the seed culture and statically incubated at 28 °C for 30 days.

Following fermentation, ethyl acetate extraction of the culture metabolites yielded a crude extract (approximately 20 g). The crude extract was solubilized in methanol and subjected to silica gel column chromatography (100–200 mesh) with gradient elution. The elution commenced with a petroleum ether-ethyl acetate system (gradient ratio ranging from 80:1 to 1:1, *v*/*v*), followed by a dichloromethane-methanol system (gradient ratio from 50:1 to 1:1, *v*/*v*). The fractions eluted with petroleum ether-ethyl acetate (8:1, *v*/*v*) were collected and reduced under vacuum to afford a solid crude product. This intermediate was further purified via Sephadex LH-20 gel column chromatography (mobile phase: 100% methanol), generating ten fractions (Fr.1 to Fr.10). Upon combination of Fr.3 and Fr.4, dissolution in methanol, and final purification by high-performance liquid chromatography (HPLC) using a methanol-water (75:25, *v*/*v*) mobile phase at 4 mL/min, the target compound chaetomugilin O (69 mg) was successfully isolated.

### 3.4. Cell Culture

The human thyroid cancer cell line CAL-62 was obtained from Procell Life Science & Technology Co., Ltd. (Wuhan, China). Cells were cultivated in RPMI 1640 medium containing 10% fetal bovine serum and 1% penicillin-streptomycin and maintained at 37 °C in a humidified atmosphere with 5% CO_2_.

### 3.5. Cell Viability Assay

The half-maximal inhibitory concentration (IC_50_) of chaetomugilin O against CAL-62 cells was determined using a Cell Counting Kit-8 (CCK-8) assay. CAL-62 cells were seeded at a density of 5 × 10^4^ cells per well in a 96-well plate and cultured at 37 °C with 5% CO_2_ until the cells reached confluence. After treatment with a series of gradient concentrations of chaetomugilin O and a DMSO control for 48 h, the old medium was aspirated. Then, 100 μL of CCK-8 solution diluted tenfold in basal medium was directly added to each well, followed by incubation at 37 °C with 5% CO_2_ in the dark for 2–3 h. The optical density (OD value) of each well was measured at a wavelength of 450 nm using a MULTISKAN MK3 microplate reader (Thermo Fisher Scientific, Milwaukee, WI, USA). The dose–response curve was fitted using GraphPad Prism 8 software based on the model (Y = Bottom + (Top-Bottom)/[1 + (IC_50_/X) ^ HillSlope], and the IC_50_ value was calculated. Experimental results are expressed as mean ± SD. Doxorubicin hydrochloride (Dox) was used as the positive control drug.

### 3.6. Reactive Oxygen Species (ROS) Assa

Intracellular ROS levels were assessed using the DCFH-DA probe. CAL-62 cells were plated in 24-well plates and cultured until reaching approximately 80% confluence. The cells were then treated with either 20 µM of the compound chaetomugilin O or DMSO (control) for 48 h. After completion of the treatment, the cells were exposed to DCFH-DA and incubated at 37 °C for 20 min. Following PBS washing, the cells were observed using a Zeiss Axio Vert. A1 fluorescence microscope (Zeiss, Oberkochen, Germany).

### 3.7. Cell Cycle Assay

CAL-62 cells were seeded in 6-well plates and cultured until reaching 80% confluency, followed by treatment with 20 µM chaetomugilin O or DMSO (control) for 48 h. After collecting the culture medium, the cells were detached using trypsin without EDTA, and the original medium was added to terminate digestion. The cell suspension was centrifuged at 1000× *g* for 5 min to pellet the cells. After washing with PBS, the cells were transferred to a 1.5 mL tube, and the supernatant was discarded. The cell pellet was resuspended in 1 mL of pre-chilled 70% ethanol and fixed at 4 °C for 30 min. Following fixation, the cells were centrifuged at 1000× *g* for 5 min and washed twice with PBS. Subsequently, 500 μL of PI staining solution (containing 50 μg/mL PI and 100 μg/mL RNase A) was added, and the cells were incubated at 37 °C in the dark for 30 min. Cell cycle distribution was analyzed using a CyFlow Ploidy Analyser flow cytometer (Sysmex Partec GmbH, Görlitz, Germany).

### 3.8. Cell Apoptosis Assay

Annexin V-FITC/PI apoptosis detection kit was employed to assess cell apoptosis. After applying the identical treatment protocol used in the cell cycle experiment, the treated cells were stained with Annexin V-FITC and propidium iodide (PI) following the manufacturer’s guidelines, followed by a 15 min incubation in the dark. Subsequenty, a CyFlow Ploidy Analyser flow cytometer (Sysmex Partec GmbH, Görlitz, Germany) was utilized to analyze the apoptotic cell ratio.

### 3.9. RNA Extraction, Library Construction and Sequencing

Cellular total RNA was extracted using TRIzol Reagent (Invitrogen, Carlsbad, CA, USA) in accordance with the manufacturer’s instructions. RNA quality was evaluated with a 5300 Bioanalyzer (Agilent Technologies, Santa Clara, CA, USA), and quantification was performed using a NanoDrop 2000 (Thermo Fisher Scientific, Waltham, MA, USA). Only high-quality RNA samples adhering to the following standards were used for library construction: OD260/280 ratio of 1.8–2.2, OD260/230 ratio ≥ 2.0, RNA Quality Number (RQN) ≥ 6.5, 28S: 18S ratio ≥ 1.0, and total RNA amount > 1 μg.

For the RNA-seq transcriptome library, 1 μg of total RNA was processed using the Illumina Stranded mRNA Prep, Ligation protocol (Illumina, San Diego, CA, USA). The procedure included eukaryotic mRNA enrichment via poly (A) tail capture using oligo (dT) magnetic beads, fragmentation in buffer, first-strand cDNA synthesis with random hexamer primers, double-stranded cDNA generation, followed by end repair, phosphorylation, and adapter ligation. Magnetic beads were used for size selection of 300–400 bp fragments, which were amplified through 10–15 PCR cycles. After quantification with a Qubit 4.0 (Thermo Fisher Scientific), the sequencing library was sequenced on the NovaSeq X Plus platform using a NovaSeq Reagent Kit (Illumina, San Diego, CA, USA).

### 3.10. Data Analysis

First, fastp (v0.23.4) software (with default parameters) was used to conduct quality assessment of the raw sequencing data, yielding clean reads. After that, HISAT2 (v2.1.1) software was employed to align the clean reads of each sample to the reference genome in strand-specific mode. Once alignment was completed, StringTie (v2.2.1) software was utilized to perform transcript assembly for each sample against the reference genome.

To identify differentially expressed genes (DEGs) among samples, transcript expression levels were computed based on TPM (Transcripts Per Million) values, and RSEM was used to quantify the abundance of gene expression. DESeq2 was applied for differential expression analysis, with the following filtering criteria adopted: |log_2_ FC| ≥ 1 and FDR < 0.05. In addition, functional enrichment analysis (including GO and KEGG analyses) was carried out on the significantly differentially expressed genes. Taking the whole transcriptome as the background, a threshold of Bonferroni-corrected *p*-value < 0.05 was used to screen out significantly enriched GO terms and metabolic pathways. Specifically, Goatools was implemented for GO enrichment analysis, whereas Python (v3.9) scipy (v1.10.1) software was used to perform KEGG pathway analysis.

### 3.11. Molecular Docking

Molecular docking was performed using AutoDock Vina 1.5.7 software [24]. The crystal structure of Akt in complex with an inhibitor (PDB: 4GV1) was obtained from the Protein Data Bank (PDB) for analysis. Using PyMOL 3.1.6.1 software, water molecules and the original co-crystallized ligand were removed from the structure, and hydrogen atoms were added to prepare the receptor file. The docking search space was defined by a grid box centered on the centroid of the original co-crystallized ligand (coordinates: x = −26.571 Å, y = 2.775 Å, z = 16.251 Å). The dimensions of the grid box were set to 40 Å × 40 Å × 40 Å to ensure comprehensive coverage of the potential binding region. The exhaustiveness parameter was set to 100. The postures generated by the docking simulations were visualized and analyzed with PyMOL molecular graphics software.

### 3.12. Statistical Analysis

Experimental data were statistically analyzed using GraphPad Prism 10. All experiments were independently repeated three times, with data presented as mean ± standard deviation (SD). Significance levels were denoted as follows: * *p* < 0.05, ** *p* < 0.01, *** *p* < 0.001, **** *p* < 0.0001; “ns” indicates *p* ≥ 0.05 (no statistically significant difference).

## 4. Conclusions

This study demonstrates that the marine fungal-derived compound chaetomugilin O inhibits the proliferation of thyroid anaplastic carcinoma CAL-62 cells through multiple pathways. Experimental results show that it reduces cell viability in a dose-dependent manner (IC_50_ = 13.57 µM), with 20 µM treatment significantly increasing intracellular ROS levels, inducing G2/M phase arrest, and promoting apoptosis. Mechanistically, transcriptomic analysis reveals significant suppression of the PI3K-Akt signaling pathway, with differentially expressed genes involved in oxidative stress, apoptosis regulation, and DNA damage repair pathways. Molecular docking confirms that chaetomugilin O targets the Akt active site with a binding energy of −8.6 kcal/mol, forming a hydrogen bond with the key residue Lys158. The compound triggers oxidative damage through ROS accumulation, synergizes cell cycle arrest with apoptosis, and interferes with the core node of the PI3K-Akt pathway, demonstrating potential to overcome thyroid cancer drug resistance. Collectively, these findings provide robust experimental evidence underpinning the use of marine natural products in targeted therapeutic strategies against thyroid cancer.

However, this study has certain limitations: the transcriptome results have not been validated by RT-qPCR for key downregulated genes, and further confirmation at the mRNA level is required. Additionally, the mechanistic investigation was solely based on the CAL-62 cell line, and validation in more thyroid cancer and other tumor cells is still needed to ascertain its applicability.

## Figures and Tables

**Table 1:** Chaetomugilin O ^1^H and ^13^C NMR data (500, 125 MHz, DMSO-*d*_6_).

Position	Chaetomugilin O	
	*δ*_C_	*δ*_H_ (*J* in Hz)
1	145.6	7.69, (s, 1H)
2		
3	157.2	
4	104.6	6.94, (s, 1H)
4a	140.5	
5	108.9	
6	184.0	
7	83.3	
8	43.4	4.29, (d, 12, 1H)
8a	113.4	
9	120.6	6.63, (d, 15.8, 1H)
10	146.0	6.72, (dd, 15.8, 7.6, 1H)
11	38.0	2.76, (m, 1H)
12	28.5	1.63, (m, 2H)
13	11.5	1.09, (t, 7.4, 3H)
7-Me	23.2	1.76, (s, 3H)
11-Me	19.2	1.28, (d, 6.65, 3H)
1′	169.8	
2′	51.3	5.37, (d, 12.05, 1H)
3′	192.8	
4′	136.8	
5′	145.3	7.30, (q, 6.90, 1H)
6′	15.1	2.09, (d, 6.85, 3H)
4′-Me	11.0	1.99, (s, 3H)

**Table 2:** IC_50_ test results of compound Chaetomugilin O.

Sample	IC_50_ (µM)
Chaetomugilin O	13.570 ± 0.170
Dox	0.167 ± 0.023

## Data Availability

The transcriptomic raw data generated in this study have been deposited in the NCBI Sequence Read Archive (SRA) under accession number PRJNA1296796. Additional data supporting the findings of this study are available from the author Y.F. upon reasonable request.

## Supplementary Materials

The following supporting information can be downloaded at: https://www.mdpi.com/article/10.3390/md23100370/s1, Figure S1: HRESIMS spectrum of chaetomugilin O. Figure S2: ^1^H NMR (500 MHz, DMSO-*d*_6_) spectrum of chaetomugilin O. Figure S3: ^13^C NMR (125 MHz, DMSO-*d*_6_) spectrum of chaetomugilin O.
