# Supplementary material for: Cytotoxic Mechanism of Deep-Sea Fungus Chaetomium globosum YP-106 Metabolite Chaetomugilin O in Thyroid Cancer Cells
Source: Mar Drugs. 2025 Sep 24;23(10):370. doi: 10.3390/md23100370 (PMC12565306; doi:10.3390/md23100370)

Supplementary Materials

Cytotoxic Mechanism of Deep-Sea Fungus Chaetomium globosum YP-106 Metabolite Chaetomugilin O in Thyroid Cancer Cells

Yaqin Fan [1,2,+], Wenhui Xiong [1,+], Yutting Qiu[1], Yang Li [1], Xin Liu [1], Peiqing He[2] and Guian Huang [1,*]

[1] School of Life Sciences, Qingdao Agricultural University, Qingdao266109, China; fanyaqin@qau.edu.cn (Y. F.); xiong0215nn@163.com (W.X.); 1260313818@qq.com (Y.Q.); 19161021606@163.com (Y.L.); liuxin202312@163.com(X.L.)

[2] MNR Key Laboratory of Marine Eco-Environmental Science and Technology, First Institute of Oceanography, Ministry of Natural Resources, Qingdao 266061, China; hepeiqing@fio.org.cn (P.H.)

* Correspondence: qdguian@sina.com

[+] These authors contributed equally to this work.

Contents

Figure S1. HRESIMS spectrum of chaetomugilin O.

Figure S2. ^{1}H NMR (500 MHz, DMSO-d_6) spectrum of chaetomugilin O.

Figure S3. ^{13}C NMR (125 MHz, DMSO-d_6) spectrum of chaetomugilin O.

Figure S1. HRESIMS spectrum of chaetomugilin O.

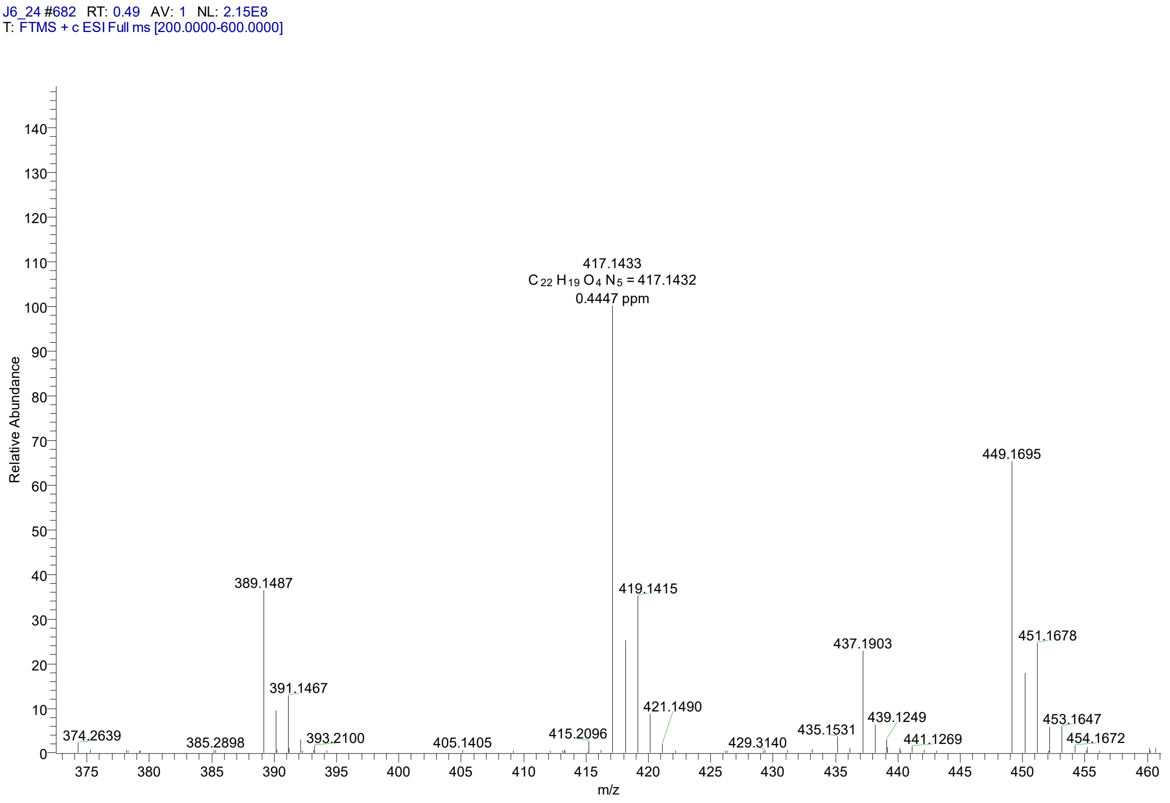

Figure S2. ^{1}H NMR (500 MHz, DMSO-d_6) spectrum of chaetomugilin O.

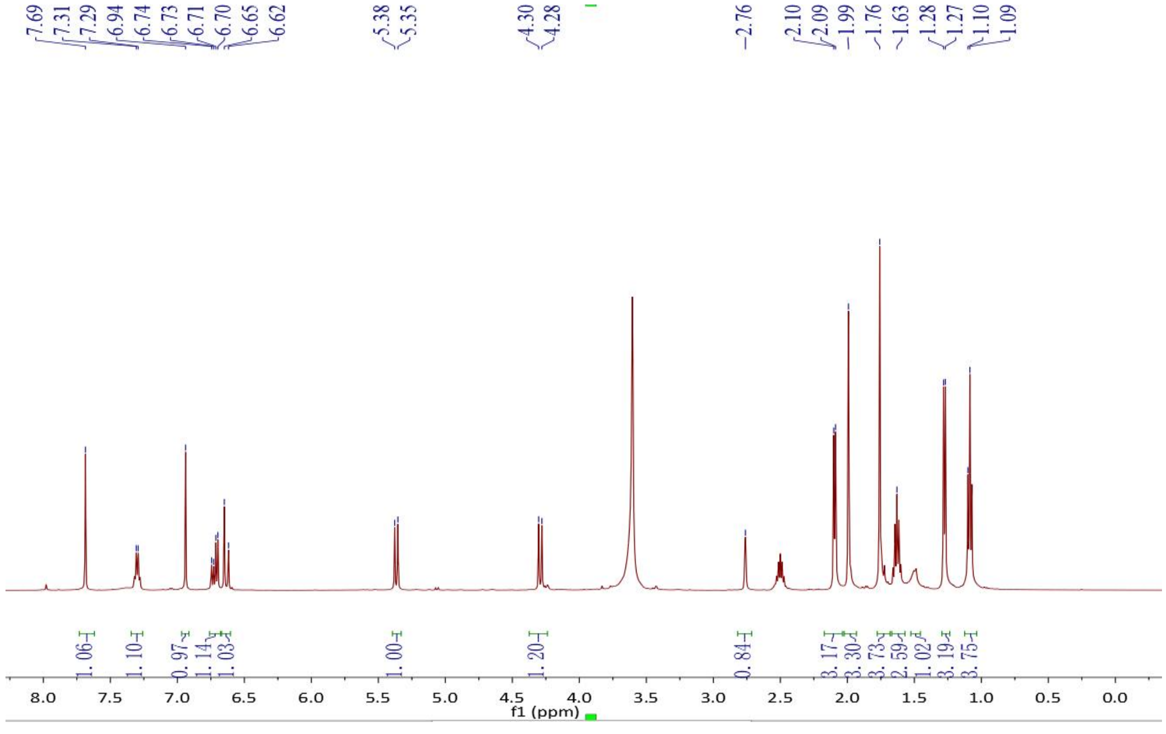

Figure S3. ^{13}C NMR (125 MHz, DMSO-d_6) spectrum of chaetomugilin O.

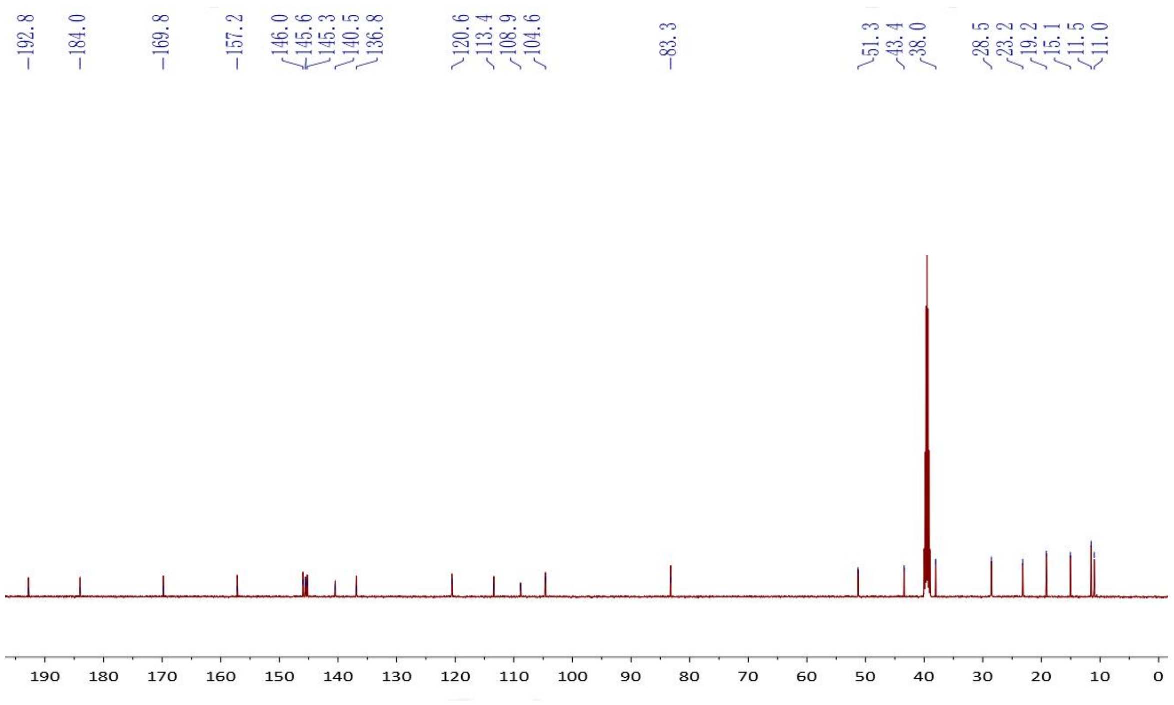

Supplement: Supplementary file 1 [file marinedrugs-23-00370-s001.zip › marinedrugs-3870548-supplementary.pdf]
